# Supplementary material for: Exploring Amodiaquine’s Repurposing Potential in Breast Cancer Treatment—Assessment of In-Vitro Efficacy & Mechanism of Action
Source: Int J Mol Sci. 2022 Sep 28;23(19):11455. doi: 10.3390/ijms231911455 (PMC9569809; doi:10.3390/ijms231911455)
Supplement: Supplementary file 1 [file ijms-23-11455-s001.zip › ijms-1916006-supplementary.pdf]

SUPPLEMENTARY DATA

Repurposing an Antimalarial Drug for Efficacy in Diverse Breast Cancer Subtypes

Detailed Methods

Cytotoxicity Studies

AQ was evaluated for its cytotoxicity efficacy in four different breast cancer cell lines: MCF-7, MDAMB-231, SK-BR3, and BT-549. Briefly, cells were grown in FBS supplemented media as described in *Cell Lines and Materials* section; and were seeded in TC treated 96-well plates (Eppendorf, Hauppauge, NY, USA) at a seeding density of 2,500 cells/well (7,500 cells/cm^2), incubated overnight for adherence at 37ºC/5% CO_2. Treatments were added next day at different AQ concentrations ranging from 0.39-50 µM. Blank culture media was added as control. After 72 hours of incubation, % cell viability was determined by performing MTT assay as described earlier [1], by reading the absorbance of dissolved formazan crystals at 570 nm (Tecan Spark 10M; Tecan, Männedorf, Switzerland).

Scratch Assay

In-vitro scratch assay was used to study the cell migration. Briefly, MDAMB-231 cells were plated in 24-well cell culture plates at a seeding density of 1×10^5 cells/well followed by overnight incubation. Next day, scratches were made along the center with a sterile 200 µL pipette tip. Thereafter, the debris was removed, and cells were washed twice with 1 mL of the PBS to assure the edges of the scratch were smoothed and then reference markings were drawn near the scratch area from the bottom side of the plate. Scratch images were captured within the marked area using inverted microscope (Laxco, Mill Creek, WA, USA) with 10X magnification. Later treatments of control, AQ (7.5, 15, 25µM) were added to the respective wells and incubated followed by imaging

after 12 hours. The captured images were analyzed quantitatively to assess the inhibitory effect of AQ on cell migration. Scratch width was measured from all wells using ImageJ software (Version 1.53c) (National Institute of Health, USA) and % scratch closure was calculated further, considering reformation of cellular monolayer as 100% scratch closure.

Clonogenic Assay

MCF-7 or MDAMB-231 cells were seeded into 6-well cell culture plates at seeding density of 500 cells per well for each cell line. Plates were kept for overnight incubation to allow cells to adhere. Next day, media was replaced, and cells were treated with AQ (7.5 µM, 15 µM and 25 µM), or control for 48 hours after which media was replaced with fresh culture medium on alternative days for 7 days. On 7th day, the colonies were stained with crystal violet as described previously [2]. After staining cells were washed with distilled water and images were captured using digital camera. Cell colonies were counted by colony counter software Open CFU [3].

3D Spheroid Study

For this study, a 3D cell-based spheroid model was developed for MCF-7 and MDAMB-231 cell lines. Briefly, MCF-7 or MDAMB-231 cells were first seeded in Corning® ultralow attachment spheroid 96-well plates (Corning, NY, USA) at a density of 5.0×10^2 cells/well and were incubated at 37°C/5% CO_2. All wells were observed for spheroid growth and images were captured on next day. Then spheroids were subjected to two kinds of dosing treatment regimens i.e., either single dose or multiple dose. Briefly, both single and multiple dosing spheroids were treated with 15, 30 and 50µM concentrations of AQ (to maintain original concentrations of 7.5, 15 and 25µM employed in the beginning), and images were captured. Images were captured on day 3, 6 and 10

following treatment. ImageJ software (Version 1.53c) (National Institute of Health, USA) was used to quantify all the spheroid images.

Supplementary Table S1: Primer sequences tested to determine the gene expression changes in response to drug treatments

S. No	Primer name	Primer Sequence
1.	POLR1A-F	TTTGCCGTGTATGGCATCGC
2.	POLR1A-R	TGTCATCTGCTGTAGCGGGG
3.	POLR1D-F	TGCACGAGGAAGACCATACCC
4.	POLR1D-R	ACAGCTGGAAGGGTACCTCG
5.	POLR1E-F	GTGACTGCTCTGGTCAGCGA
6.	POLR1E-R	ACACGTCTTCAGGCTTGGCT
7.	TAF1B-F	CCCCGTCAGCAAAGCATCAC
8.	TAF1B-R	GGCAAGTGTCTGTGGCATGG
9.	LAMP1-F	ATGTGTTAGTGGCACCCAGG
10.	LAMP1-R	TGTTCACAGCGTGTCTCTCC
11.	CDKN1A-F	AATCGTCCAGCGACCTTCCT
12.	CDKN1A-R	CTGACTCCTTGTTCCGCTGC
13.	GADD34-F	TGAGTCAGACCCACATCCCT
14.	GADD34-R	TGGCCTTTAGGGGAGTCTCA
15.	DDIT3-F	TTCTCTGGCTTGGCTGACTG

16.	DDIT3-R	TTCCTGCTTGAGCCGTTCAT
17.	BNIP3-F	CTTTAAACACCCG AAGCGCA
18.	BNIP3-R	GGAGGTTGTCAGACGCCTT
19.	BNIP3L-F	AGAAAGTAGAAA CCCGTGGGAG
20.	BNIP3L-R	ATCTTCTTGTGGCGAAGGGC

S. No	Primer name	Primer Sequence
21.	Noxa-F	TACCGCTGGCCTACTGTGAA
22.	Noxa-R	ATGTGCTGAGTTGGCACTGA
23.	LC3-II-F	CAGCGTCTCCACACCAATCT
24.	LC3-II-R	TCTCCTGGGAGGCATAGACC

References

[1] N.S. Kulkarni, V. Parvathaneni, S.K. Shukla, L. Barasa, J.C. Perron, S. Yoganathan, A. Muth, V. Gupta, Tyrosine kinase inhibitor conjugated quantum dots for non-small cell lung cancer (NSCLC) treatment, Eur. J. Pharm. Sci. Off. J. Eur. Fed. Pharm. Sci. 133 (2019) 145–159. https://doi.org/10.1016/j.ejps.2019.03.026.

[2] V. Parvathaneni, N.S. Kulkarni, S.K. Shukla, P.T. Farrales, N.K. Kunda, A. Muth, V. Gupta, Systematic Development and Optimization of Inhalable Pirfenidone Liposomes for Non-Small Cell Lung Cancer Treatment, Pharmaceutics. 12 (2020). https://doi.org/10.3390/pharmaceutics12030206.

[3] Q. Geissmann, OpenCFU, a New Free and Open-Source Software to Count Cell Colonies and Other Circular Objects, PLOS ONE. 8 (2013) e54072. https://doi.org/10.1371/journal.pone.0054072.
